# Quantitative polymerase chain reaction analysis by deconvolution of internal standard

**DOI:** 10.1186/1471-2199-11-30

**Published:** 2010-04-29

**Authors:** Yasuko Hirakawa, Rheem D Medh, Stan Metzenberg

**Affiliations:** 1 Department of Biology, California State University, 18111 Nordhoff St. Northridge, California 91330, USA

## Abstract

**Background:**

Quantitative Polymerase Chain Reaction (qPCR) is a collection of methods for estimating the number of copies of a specific DNA template in a sample, but one that is not universally accepted because it can lead to highly inaccurate (albeit precise) results. The fundamental problem is that qPCR methods use mathematical models that explicitly or implicitly apply an estimate of amplification efficiency, the error of which is compounded in the analysis to unacceptable levels.

**Results:**

We present a new method of qPCR analysis that is efficiency-independent and yields accurate and precise results in controlled experiments. The method depends on a computer-assisted deconvolution that finds the point of concordant amplification behavior between the "unknown" template and an admixed amplicon standard. We apply the method to demonstrate dexamethasone-induced changes in gene expression in lymphoblastic leukemia cell lines.

**Conclusions:**

This method of qPCR analysis does not use any explicit or implicit measure of efficiency, and may therefore be immune to problems inherent in other qPCR approaches. It yields an estimate of absolute initial copy number of template, and controlled tests show it generates accurate results.

## Background

Quantitative polymerase chain reaction (qPCR) is a collection of methods for measuring amounts of specific template DNA sequences [1,2]. In one approach the binding of a reporter dye (SYBR^® ^Green I) to double-stranded DNA is used to quantify the progress of the PCR at each cycle of synthesis, and a pattern resembling exponential or sigmoidal growth is recorded until the fluorescence reaches a plateau level [3,4]. The principle of quantification is that reactions initiated with fewer DNA copies require more cycles of replication to achieve a product yield, just as runners starting a race from a greater distance require more steps to reach a finish line. However, in the absence of information on average length of stride it is impossible to know the starting line of a running race from the number of steps taken. Extant qPCR methods are similarly flawed because the reaction efficiencies and initial template amounts cannot be simultaneously deduced [5,6].

In one simple mathematical model, the instantaneous rate of exponential growth of fluorescence is measured in the earliest cycle possible, when the logarithm of fluorescence is still linearly related to cycle number, and it is assumed that reaction efficiency (**Φ**) has been constant to that point [7-9]. The product fluorescence (**R**_**n**_) is related to the starting amount of template (**R**_**0**_) by **R**_**n **_= **R**_**0**_**Φ**^**n **^where 1 <**Φ **≤ 2 and **n **is the number of cycles. A different and widely used approach for estimating efficiency is to prepare a set of dilutions of a starting template and to measure the respective cycle numbers (**c**_**q**_) at which each sample reaches a threshold level of fluorescence [10]. Theoretically if product consistently doubles in each PCR cycle, then halving the starting template in a sample requires that one extra cycle be added to achieve the same ending yield. If reaction efficiency is constant in each cycle and sample, its value (**Ψ**) is calculated from the equation **Ψ **= 10^-1/**s**^where **s **is the slope of the linear relationship **c**_**q **_= **s **log_10_(dilution) + **b**. The relative starting amount is again deduced by assuming exponential growth **R**_**n **_= **R**_**0**_**Ψ**^**n**^. A third model is based on the assumption that PCR efficiency is not constant, but decreases as a logistic function of the yield [11-13]. The growth of product is described by the function **R**_**i **_= **R**_**max**_(1+*e*^**(m-i)/k**^)^-1 ^where **R**_**i **_is the fluorescence yield at the end of cycle **i**, **R**_**max **_is the plateau fluorescence, **m **is the cycle number at which **R**_**m **_= **R**_**max**_/2, and **k **is a curve fitting constant related to initial reaction efficiency. Extrapolation of the sigmoidal curve back to the zero cycle is used to estimate starting template **R**_**0 **_= **R**_**max**_(1+*e*^**m/k**^)^-1^.

We demonstrate in this paper a new method of qPCR analysis that is independent of any explicit or implicit mathematical model for efficiency, and thereby overcomes the fundamental problem of current methods of analysis [14,15]. The qPCR is standardized by admixture of the amplicon product as an internal benchmark, and a computational algorithm is applied to determine the absolute amount of the "unknown" component in a reaction by deconvolution. In control experiments with known standards the method was accurate and precise, estimating 103% (± 9.8% s.d., n = 6) of the true number of copies. We show that the method is superior to other methods of analysis using the same control data, and apply the new method to demonstrate the induction of human *RCAN1.1 *and B-cell translocation gene 1 (*BTG-1*) transcripts in acute lymphoblastic leukemia cell lines following treatment with dexamethasone.

## Methods

### Control DNA templates and oligonucleotide primers

PCR (for preparation of control template) and qPCR were conducted using these primer sets: RCAN1.1 (96 bp amplicon), 5'-ACCATCGCCTGTCACCTGGA and 5'-GGTGATGTCCTTGTCATACGTCCT; BTG-1 (130 bp amplicon), 5'-AGGCTTCTCCCAAGTGAACT and 5'-TTGGTGCTGTTTTGAGTGCT; E4BP4 (250 bp amplicon), 5'-ATGGGGAATTCTTTCTCTGG and 5'-CTTTGATCCGGAGCTTGTGT; β-actin (130 bp amplicon), 5'-AGTCCTCTCCCAAGTCCACA and 5'-CACGAAGGCTCATCATTCAA; β-actin (285 bp amplicon, alternatively spliced), 5'-TCATGAAGTGTGACGTTGACATCCGT and 5'-CTTAGAAGCATTTGCGGTGCACGATG. Control templates for β-actin, RCAN1.1, and E4BP4 were established by PCR from CEM-C7-14 cells [16]. A BTG-1 control DNA was established by PCR from a cloned mouse cDNA of *BTG-1*, kindly provided by Dr. Sang Geon Kim (Seoul National University, South Korea). Control PCR templates were purified by polyacrylamide gel electrophoresis in 1× TAE buffer, and the DNA eluted from a crushed slice into 100 mM NaCl, 10 mM Tris-Cl, 0.5 mM EDTA, pH 8.0. The DNA control was collected from the eluate by ethanol precipitation, resuspended in sterile deionized water and quantified by spectrophotometry at 260 and 280 nm. The DNA was diluted in sterile water using calibrated micropipettes, for use as an admixed standard in efficiency-free qPCR assays. The equipment and materials used during preparation of these DNA standards should be decontaminated to prevent cross-contamination during reaction assembly.

### qPCR reaction assembly

The qPCR reaction is assembled with 1 μl of an "unknown" DNA sample (diluted 1x, 0.5x, 0.25x, 0.125x or 0.0625x), 1 μl of sterile water or a known amount of control DNA (e.g. 250,000 copies) of the same amplicon being tested, 1 μl of each oligonucleotide primer (typically 5-10 μM), and 12.5 μl of SYBR^® ^JumpStart™ Taq ReadyMix (Sigma-Aldrich) in a 25 μl reaction. The qPCR was conducted in an Applied Biosystems 7300 Real-Time PCR System using a program of 94°C, 2 min, and 20-45 cycles of (94°C, 30 sec; 57°C, 45 sec; 72°C, 60 sec), except for the E4BP4 amplicon in which 60°C was the annealing temperature.

### Computational deconvolution

The open-source perl program Deconvolution is available (Additional file 1), along with a sample template file (Additional file 2) and program guide (Additional file 3). In brief, the algorithm is based on optimization of the linear correlation between log_10_(**A**_**0**_) and **c**_**q**_, where **A**_**0 **_is the starting template copies built from a linear combination of "known" (**N**) and "unknown" (**U**) components. The known amount **N **is an internal standard, prepared from a gel-purified PCR product, and **U **is a measured amount of an experimental sample of unknown concentration. The value of **U **is deduced through the optimization of the aforementioned correlation.

The program user sets a range of estimated values of **U **(the operating program offers a suggested range, by default) and range of fluorescence threshold values, and the number of steps to use in dividing each range. An optimization is conducted simultaneously over 50 to 100 iterations of **U **and fluorescence threshold (i.e. 2500 to 10000 separate determinations of the correlation coefficient across all samples). The value of **U **giving the optimal correlation (R^2^) is the putative value of the 1x "unknown". At a conceptual level, the deduced value of **U **reflects an underlying assumption that the known and unknown components in the reaction are amplified with the same geometric mean efficiency (defined as **E **= (**A**_**n**_/**A**_**0**_)^1/**n **^where **A**_**n **_is the template copies at the end of cycle **n**).

### Application of method to cDNA templates

The growth and propagation of cell lines CEM-C7-14 and CEM-C1-15 cells, dexamethasone treatment, extraction of cellular RNA and cDNA preparation are described by Priceman, et al. [17]. In this study, first-strand cDNA synthesis was conducted for 3 h at 42°C in a reaction volume of 25 μl with 5 μg of total cellular RNA and an oligo(dT) primer. The cDNA product was diluted for use in qPCR experiments, such that 1 μl of the cDNA reaction (from 200 ng of total RNA) represented a "1x" (**f**_**i **_= 1.0) amount of unknown template.

### Comparative methods of qPCR analysis

The LinReg method was implemented using the software package LinReg 11.1 [9]. Linear Regression of Efficiency (LRE) was implemented using the Java program LRE Analyzer [11], and **R**_**0 **_(or **F**_**0**_) values were determined from the instrument calibration described below. RCAN1.1 and β-actin amplifications were based on differing initial primer concentrations (500 nM and 250 nM, respectively).

### Calibration of qPCR instruments for measurement of geometric mean efficiencies

Instrument calibration is not required for the method described in this paper; however, we have determined the relationship between fluorescence and double stranded DNA concentration on our Applied Biosystems 7300 Real-Time PCR Systems to understand why certain extant methods of qPCR analysis do not yield correct results. The calibrations of the instruments were established by polyacrylamide gel electrophoresis and optical densitometry of ethidium bromide stained qPCR product (with known instrument fluorescence); the gel staining was calibrated using known amounts of φ x174/HaeIII fragments. The standard deviation propagated by calculating DNA mass equivalence from SYBR^® ^Green I fluorescence was approximately 10%, based on variances in slope and y-intercept of the two standard curves (ng φ x174 fragments to gel fluorescence, n = 18; and qPCR gel fluorescence to SYBR Green I instrument fluorescence, n = 5).

## Results

### An efficiency-independent method of determining copy number

We developed a method of qPCR analysis for estimating the absolute amount of template in a sample by a software-assisted optimization process. A sample of DNA with an unknown template copy number (**U**) is used in a series of dilutions that include known amounts of the same template sequence:

where **f**_**i **_is the dilution factor of the unknown template in PCR well **i **(*e.g. ***f**_**i **_= 1.0, 0.5, 0.25, 0.125, ...), **N**_**i **_is a known amount of the template sequence (*e.g. ***N**_**i **_= 0, 10000, 50000 DNA copies, ...), and their sum **A**_**0, i **_is the overall initial template copies. We typically set up experiments with 10 samples, of which four have admixed internal control (**N**_**i **_> 0). The qPCR is conducted and threshold or quantification cycle values **c**_**q **_are determined over a span of fluorescence thresholds, typically 50 to 100 logarithmically distributed values (**T**_**k**_) between 1% and 10% of the plateau fluorescence. A span of 50 to 100 assumed values for the unknown template component (**U**_**j**_) is also established over a several-log range, and used pair-wise with each threshold fluorescence to solve for the linear regression correlation (R^2^) of **c**_**q **_as a function of log_10_(**f**_**i**_**U**_**j **_+ **N**_**i**_). This is initially conducted over approximately 2500 paired values of **U**_**j **_and **T**_**k**_, and may be iterated over a more narrow range to any desired level of precision. Ultimately the value **U**_**j **_that optimizes R^2 ^represents a deconvolution of **A**_**0 **_into "known" **N**_**i **_and unknown **U **values.

**Figure 1:**
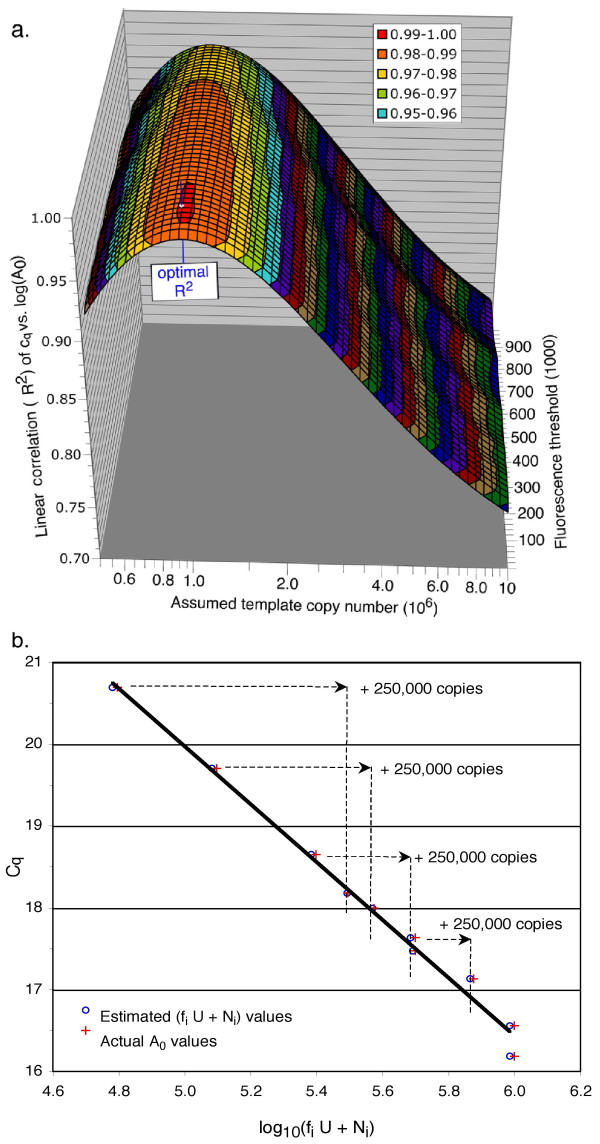
**Determination of initial copy number of RCAN1.1 control DNA by efficiency-independent qPCR**. (a) Linear correlations of **c**_**q **_*vs. *log_10_(**f**_**i**_**U**_**j **_+ **N**_**i**_) are shown as a function of the assumed template copy number (**U**_**j**_, on a logarithmic axis), and fluorescence threshold (**T**_**k**_). Colors are keyed to indicate 0.01 unit steps of R^2^, and the optimal correlation (R^2 ^= 0.995, **U **= 9.91 × 10^5 ^copies, **T **= 1.14 × 10^4 ^fluorescence units) was identified by iteration over 100 estimates (5 × 10^5 ^≤ **U**_**j **_≤ 1 × 10^7^), and 50 thresholds (10^4 ^≤ **T**_**k **_≤ 10^6^) where **R**_**max **_= 2.47 × 10^6 ^fluorescence units. (b) Graph of **c**_**q **_*vs. *log_10_(**f**_**i**_**U **+ **N**_**i**_) representing the optimal estimate **U **from the same experiment, which was conducted using 10 samples (replicate dilutions of **f**_**i **_= 1.0, 0.5, 0.25, 0.125, and 0.0625, with **N**_**i **_= 2.5 × 10^5 ^amplicon copies added to four samples, as indicated by the dashed lines. Graph line: **c**_**q **_= -3.53 log_10_(**f**_**i**_**U **+ **N**_**i**_) + 37.6 (R^2 ^= 0.995).

Figure 1a shows the results of a qPCR using the new method, with dilutions of 1 × 10^6 ^copies of a human *RCAN1.1 *DNA as a control template. The method generated a predicted value of **U **= 9.91 × 10^5 ^copies (99.1% of the true copy number), at an optimal correlation coefficient R^2 ^= 0.995. The graph of **c**_**q **_vs. log_10_(**f**_**i**_**U **+ **N**_**i**_) representing the optimal value of **U **is shown in figure 1b, and both estimated (o) and actual (+) copy numbers are shown. The optimization over **U **is largely independent of fluorescence threshold, as evidenced by the broad "spine" of the peak in figure 1a, and is valid even outside of the linear range of detection of the instrument.

**Figure 2:**
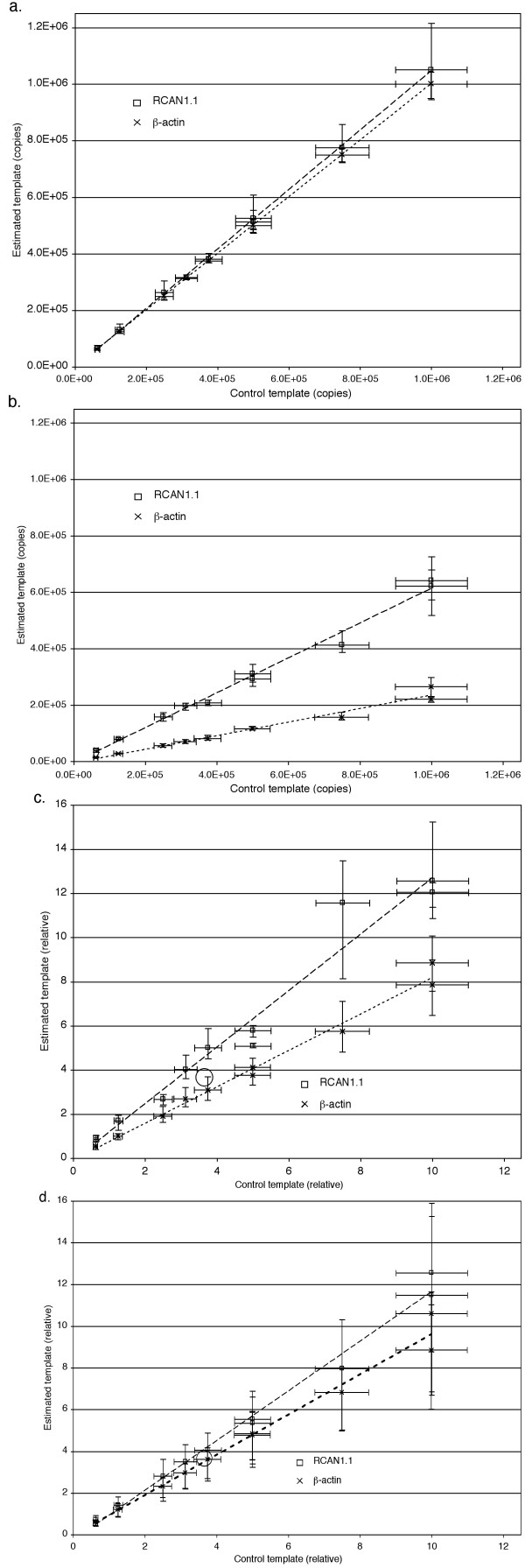
**Estimated vs. actual template copy numbers, applying the same data to four methods of analysis**. Symbols are mean values for each amplicon (n = 3, 10 samples each as in Figure 1; y-axis error bars, minimum and maximum copy number estimates; x-axis error bars, 10% s.d. from *a priori *error in pipetting and dilution). (a) Efficiency-independent method described in this paper (absolute quantification). RCAN1.1 dashed line (y = 1.05x - 5180, R^2 ^= 0.9996), β-actin dotted line (y = 1.00x - 83, R^2 ^= 0.9999). (b) Linear Regression of Efficiency (LRE) method (absolute quantification). Average correlations of fluorescence data to sigmoidal curves: R^2 ^= 0.9987 (n = 30, RCAN1.1, E_max _= 0.912 ± 0.007 s.d.) and R^2 ^= 0.9999 (n = 30, β-actin, E_max _= 0.941 ± 0.007 s.d.). RCAN1.1 dashed line (y = 0.618x - 4790, R^2 ^= 0.9903), β-actin dotted line (y = 0.240x - 5650, R^2 ^= 0.9751). (c) LinReg method (relative quantification). Y-axis is scaled to the geometric mean of data (open circle). Average correlations (log_10_**R**_**n **_*vs. ***n**, over 4 cycles) for determination of **Φ**: R^2 ^= 1.000 (n = 30, RCAN1.1) and R^2 ^= 1.000 (n = 30, β-actin). RCAN1.1 dashed line (y = 1.28x - 0.0951, R^2 ^= 0.9601), β-actin dotted line (y = 0.825x - 0.0761, R^2 ^= 0.9880). (d) Serial dilution method (relative quantification). Y-axis is scaled to the geometric mean of data (open circle). Average graph correlations (**c**_**q **_*vs. *log_10_**A**_**0**_) for determination of **Ψ**: R^2 ^= 0.9959 (RCAN1.1) and R^2 ^= 0.9947 (β-actin). RCAN1.1 dashed line (y = 1.19x - 0.261, R^2 ^= 0.9888), β-actin dotted line (y = 0.965x - 0.048, R^2 ^= 0.9820).

In six control amplifications similar to that shown in figure 1a (three with the 96 bp RCAN1.1 amplicon and three with a 130 bp amplicon of human *β-actin*), the average result was 103% (± 9.8% s.d., n = 6 experiments of 10 samples each) of the spectrophotometrically determined value of **U **(figure 2a). The data from these six control experiments were also analyzed by three popular methods of qPCR analysis (figures 2b, c, and 2d). The Linear Regression of Efficiency (LRE) method underestimated the copy numbers of RCAN1.1 and β-actin by 38% and 76%, respectively (figure 2b). Analysis of the logarithmic graph of fluorescence as a function of cycle number was also performed using the software package LinReg (figure 2c), and taken together the data from the six control experiments (n = 60 samples) show good proportionality between the estimated and known amounts. However, taken separately the RCAN1.1 and β-actin experiments generated estimates using LinReg that were 1.28 and 0.82 times their expected values, and the triplicate data points showed an average standard deviation of 16%. The observed overestimation of initial RCAN1.1 template in these experiments is a consequence of an underestimation of efficiency (**Φ **<**E**), and conversely **Φ **>**E **in the β-actin results. Estimates of relative initial copy number were also calculated (figure 2d) by the method of Pfaffl, using values of **Ψ **established from the slopes of the graphs of **c**_**q **_vs. log_10_(**A**_**0**_). These also showed good proportionality between the estimated and known amounts as a group, but taken separately the RCAN1.1 and β-actin results generated estimates that were respectively 1.19 and 0.96 times their expected values, and the triplicate data points showed an average standard deviation of 29%. Overestimation of initial RCAN1.1 template is again a consequence of an underestimation of efficiency (**Ψ **<**E**).

**Figure 3:**
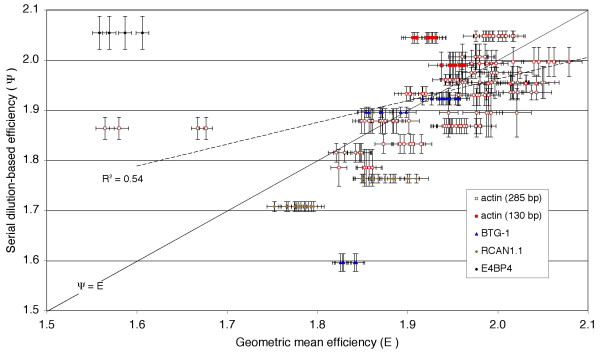
**Geometric mean efficiency (E) is not revealed by serial dilution-based determinations of Ψ**. Symbols indicate 219 individual samples, from 28 dilution experiments involving 5 different amplicons. Y-axis error bars are the standard deviations of **Ψ **(propagated from the slope variance of each experimental **c**_**q **_*vs. *log_10_**A**_**0 **_graph); x-axis error bars are standard deviations of **E **= (**A**_**Cq**_**/A**_**0**_)^1/Cq ^where **A**_**Cq **_has a known *a posteriori *error of 8% (from variance in qPCR instrument calibration slopes), and **A**_**0 **_has an *a priori *error of 10% (from known precision of pipetting and dilution). Dashed diagonal line: Linear regression correlation of **Ψ ***vs. ***E **(R^2 ^= 0.54). Solid diagonal line: Reference along which **Ψ **= **E**.

Figure 3 shows that in actual controlled experiments the slope-based efficiency **Ψ **is only weakly related (R^2 ^= 0.54) to the true efficiency **E**, even when limited to 28 control experiments (219 samples) that yielded high correlation coefficients (0.9864 ≤ R^2 ^≤ 0.9993) for the graphs of **c**_**q **_as a function of log_10_(**A**_**0**_). The solid diagonal line provides a reference for the result **Ψ **= **E**, but many of the data points lie well above or below indicating that **Ψ **over- or underestimates **E **by an average of about 4%.

A mistaken assumption that **E **= **Ψ **results in a substantial error in estimation of relative copy numbers between samples. For example, if **E **= **Ψ **were assumed for the purposes of calculation then 40 of our 71 samples with the highest correlation coefficients (R^2 ^> 0.995) would have had an overestimation of yield (measured **Ψ**^**Δc **^> actual **E**^**Δc**^) averaging 1.92-fold (± 3.06) over **Δc **= 10 cycles. In a larger collection of 385 control samples with a lower correlation coefficient (R^2 ^> 0.951), the compounded error of assuming **E **= **Ψ **is more severe: 225 samples would have led to an average overestimation error (measured **Ψ**^**Δc **^> actual **E**^**Δc**^) averaging 3.7-fold (± 10) over **Δc **= 10 cycles. This result shows that **Ψ **is not generally predictive of **E **in controlled experiments.

### Application of the efficiency-independent method to quantification of cDNA

**Figure 4:**
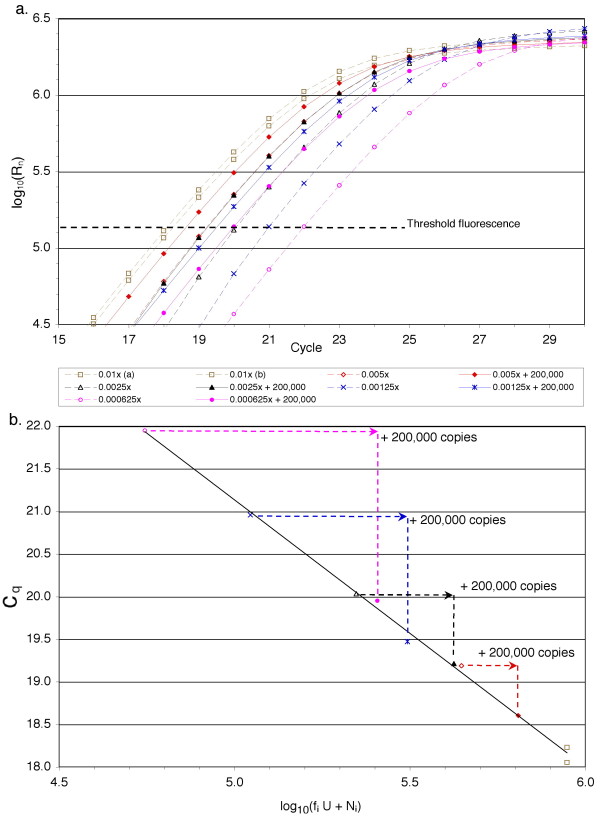
**Determination of β-actin cDNA copy number by efficiency-independent qPCR**. (a) Amplification of 130 nt β-actin amplicon from RNA of Dex-treated CEM-C1-15 cells. The samples were either neet (**N**_**i **_= 0, dashed lines) or had admixed β-actin amplicon (**N**_**i **_= 2 × 10^5^, solid lines). (b) Graph of **c**_**q **_*vs. *log_10_(**f**_**i**_**U **+ **N**_**i**_) representing the optimal estimate **U **from the same experiment. Solid line: Linear regression solution y = -3.134x + 36.8 (R^2 ^= 0.9963), from the optimal **T **= 1.34 × 10^5 ^(marked threshold in part a) and **U **= 8.9 × 10^7 ^(β-actin template copy number derived from 200 ng poly(A)^+ ^cellular RNA).

The efficiency-free qPCR method was applied to cDNA templates derived from the acute lymphoblastic leukemia cell lines CEM-C7-14 and CEM-C1-15, with and without prior growth in dexamethasone (DEX). A representative experiment is shown in figure 4a, where RNA was extracted from Dex-treated CEM-C1-15 cells and used to prepare cDNA for qPCR analysis of a 130 bp amplicon from the human *β-actin *gene. This amplification was conducted in the presence (solid lines, filled symbols) or absence (dashed lines, open symbols) of **N**_**i **_= 200,000 copies of the purified amplicon product as an admixed standard, and with **f**_**i **_varying from 0.01 to 0.000625. The optimal linear correlation between **c**_**q **_and log_10_(**f**_**i**_**U **+ **N**_**i**_) occurred at **U **= 8.9 × 10^7 ^copies β-actin template in the undiluted cDNA (R^2 ^= 0.9963). The effect of adding 200,000 copies of the purified amplicon to four of the samples is indicated graphically in figure 4b by a shift of data points to the right (to higher **A**_**0**_) and down (to lower **c**_**q**_) along the best-fit correlation line.

As shown in Table 1, the efficiency-independent method showed that DEX induction of CEM-C1-15 cells resulted in a 6.6-fold (± 0.40) increase in BTG1 cDNA and a 5.1-fold (± 0.40) increase in RCAN1.1 cDNA, normalized to levels of β-actin. The induction of CEM-C7-14 cells with DEX resulted in significantly larger increases in copy number, with a 23-fold (± 0.22) increase in BTG1 and 14-fold (± 0.41) increase in RCAN1.1 cDNA normalized to β-actin cDNA. These findings are consistent with other studies of DEX induction of these cells, and confirm that BTG1 and RCAN1.1 are differentially induced [16,17].

**Table 1:** Induction of BTG-1 and RCAN1.1 upon DEX treatment of CEM cells

	CEM-C1-15 cells		CEM-C7-14 cells	
	
Gene (Method)(*)	- DEX	+ DEX	- DEX	+ DEX
BTG-1 (Absolute)	6.5 × 10^4 ^copies (± 7.3 × 10^3^) n = 2	4.4 × 10^5 ^copies (± 1.4 × 10^5^) n = 3	4.9 × 10^4 ^copies (± 1.2 × 10^3^) n = 2	7.1 × 10^5 ^copies (± 1.2 × 10^5^) n = 2
β-actin (Absolute)	1.4 × 10^8 ^copies (± 1.4 × 10^7^) n = 2	1.4 × 10^8 ^copies (± 2.7 × 10^7^) n = 3	1.3 × 10^8 ^copies (± 1.6 × 10^7^) n = 2	8.5 × 10^7 ^copies (± 5.8 × 10^6^) n = 2
BTG-1 (Relative)	0.047% of actin (± 0.0072%)	0.31% of actin (± 0.12%)	0.037% of actin (± 0.0044%)	0.84% of actin (± 0.16%)
BTG-1 (Normalized)	1x	6.6x (± 0.40)	1x	23x (± 0.22)

RCAN1.1 (Absolute)	1.4 × 10^5 ^copies (± 2.7 × 10^3^) n = 2	5.1 × 10^5 ^copies (± 4.5 × 10^3^) n = 2	2.0 × 10^5 ^copies (± 1.8 × 10^4^) n = 2	1.3 × 10^6 ^copies (± 3.0 × 10^4^) n = 2
β-actin (Absolute)	1.0 × 10^8 ^copies (± 2.5 × 10^7^) n = 2	7.3 × 10^7 ^copies (± 2.3 × 10^7^) n = 2	1.9 × 10^8 ^copies (± 3.0 × 10^6^) n = 2	8.5 × 10^7 ^copies (± 3.4 × 10^7^) n = 2
RCAN1.1 (Relative)	0.14% of actin (± 0.034%)	0.69% of actin (± 0.22%)	0.11% of actin (± 0.010%)	1.5% of actin (± 0.61%)
RCAN1.1 (Normalized)	1x	5.1x (± 0.40)	1x	14x (± 0.41)

(*) Average copy numbers (± s.d.) of template in cDNA generated from 200 ng poly(A)^+ ^RNA. Relative copy numbers are given with respect to the 130 bp β-actin amplicon. Normalized values of BTG-1 and RCAN1.1 indicate the consequences of DEX treatment.

## Discussion

This new method of qPCR analysis does not require any assumption or calculation of PCR efficiency. The deduced concentration of unknown template (**U**) represents a point at which the "unknown" and "known" parts of the sample are concordant in amplification activity. The "known" part of the sample is a trace amount of amplicon of the same type being amplified from the "unknown" DNA component, and its presence in approximately half of the sample tubes provides an internal reference that anchors the **c**_**q **_vs. log_10_(**f**_**i**_**U **+**N**_**i**_) graph (figures 1b and 4b). As a matter of experimental design, the amount of admixed amplicon (**N**_**i**_) should be sufficient to shift the value of **c**_**q **_by a significant amount (*e.g. *1 to 3 cycles), but should not be added in such excess that **f**_**i**_**U **+ **N**_**i **_≈ **N**_**i**_. The internal DNA standard in this method is exactly the same as the amplicon product being studied, unlike previous uses of internal competitive standards in which the experimental and control amplicons differ [18-22], and this is important for assuring identical behavior in amplification. We tested the method under a wide range of primer concentrations (50 - 800 nM) and noted a decrease in **E **when very high or very low concentrations of primer were used (data not shown). However, running the reactions under non-optimal conditions did not skew the quantitative result, probably because the "known" and "unknown" parts of the sample are inhibited identically. We have not tested the method with extremely low copy numbers of template.

We have highlighted problems of accuracy with three other commonly applied methods of qPCR analysis that involve fitting data to a mathematical model. The error in use of the linear regression method (LinReg, figure 2c) highlights the difficulty of extrapolating exponential data back to the zero cycle to estimate **R**_**0 **_[23]. The geometric mean amplification efficiency is not easily predictable from a linear regression analysis of log rates in later cycles, just as the average speed of a runner is not predictable from instantaneous speeds measured at the point of crossing a finish line. In our controlled experiments with known amounts of starting template, and using the recommended software package for analysis, this method misestimated the true relative amounts of template by 18 to 28%.

The mathematical treatment applied in the LRE method (figure 2b) assumes conformity of the PCR fluorescence profile to the classic Boltzmann sigmoid function [11]. However, the fluorescence plateau that anchors this sigmoidal curve in PCR may sometimes be caused by a limitation in SYBR^® ^Green I dye fluorescence measurement, rather than a true maximal product yield, and such an artifact could skew calculations of cycle efficiency. We have found through follow-up gel electrophoresis studies that product continues to accumulate during the "plateau" phase of qPCR, at least with our instruments, particularly when primer concentrations exceed 200 nM. This suggests that **R**_**max **_values from our machine data do not represent actual maximum product yields, and there could be a proportional underestimation of **R**_**0 **_through the Bolzmann equation as well as biased values of **m **(the cycle at which a **R**_**max**_/2 is achieved) and the fitting constant **k**. This constant **k **is related to the initial efficiency of the reaction, which can be demonstrated mathematically from the derivative of fluorescence yield ***R***_**i**_' = **R**_**max **_(1+*e*^**(m-i)/k**^)^-2^*e*^**(m-i)/k **^**k**^-1 ^from which it follows that ***R***_**i**_' = ***R***_**i **_(**k**(1-*e*^**(i-m)/k**^))^-1 ^and ***R***_**i**_'_**(i = 0)**_/***R***_**i(i = 0) **_= (ln ***R***_**i**_)'_**(i = 0) **_= (**k**(1-*e*^**-m/k**^))^-1 ^≈ 1/**k **(because *e*^**-m/k **^« 1 if **R**_**max**_**/R**_**0 **_» 1000). However, (ln ***R***_**i**_)' is an instantaneous measure of log efficiency at the **i**^th ^cycle, so 1/**k **closely approximates the natural log of initial reaction efficiency. Differences in amplification curve shapes and the clipping of plateau fluorescence could be problematic for LRE, or any method that assumes conformity of the PCR fluorescence profile to the Boltzmann sigmoid function, and an erroneous estimation of initial reaction efficiency (*e*^1/**k**^) could explain the bifurcation of RCAN1.1 and β-actin graphs in figure 2b and the miscalculation of initial template copies. While we were unable to achieve success with the LRE method, others have validated the LRE and sigmoidal curve fitting methods with controls and their success may have depended on using different reaction components and/or instrumentation.

**Figure 5:**
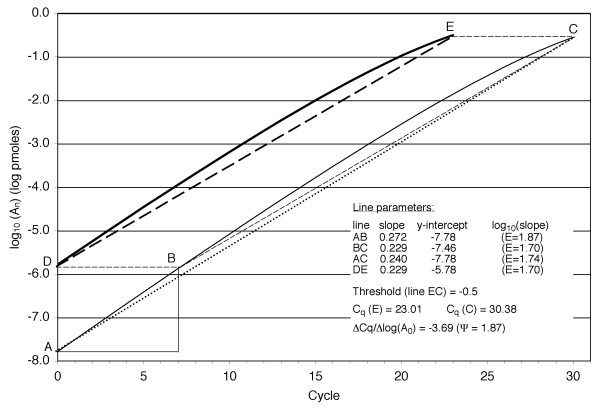
**Geometric model explanation for the differing values of Ψ and E**. If two sample amplifications are exponential with the same constant efficiency, then **Ψ **= **E **because the dilution factor (segment DA) and cycle spacing Δ**c**_**q **_(segment EC) predict the slopes in the trapezoidal figure. However, if the efficiencies drop steadily with cycle number (solid lines) then dilution can result in an increase in geometric mean efficiency (AC has a greater slope than DE). Note that **Ψ **= 1.87 is established and preserved after 7 cycles, as the spacing DB = EC, and overestimates both **E**_**DE **_= 1.70 and **E**_**AC **_= 1.74. The modelled synthesis of product is determined by a recursive function **A**_**n **_= **A**_**n-1**_(1 + **α**_**n**_**X**_**n**_) where **A**_**n **_represents the concentration of two complementary strands at the end of cycle **n**, **α**_**n **_= **K**_**A**_(**P**_**n-1**_)^2^/(1 + **K**_**A**_(**P**_**n-1**_)^2^) is the primer-annealed fraction and **X**_**n **_= log_10_(**A**_**n-1**_)/(log_10_(**A**_**n-1**_) + **I**_1/2_) is the fraction extended by polymerase. **P**_**n **_is the concentration of each primer at the end of cycle **n **(assumed to be consumed at the same rate, and initiated with **P**_**0 **_= 2.5 pmoles), **K**_**A **_= 50 pmole^-2 ^is an annealing constant, **I**_1/2 _= -1 is an inhibition constant, and the reaction volume unit arbitrarily set to 1. A and D on the ordinate axis represent log_10_(**A**_**0**_) = -7.778 (10^4 ^copies) and log_10_(**A**_**0**_) = -5.778 (10^6 ^copies), respectively, and the threshold line EC has log_10_(**A**_**n**_) = -0.5 (1.9 × 10^11 ^copies).

The third efficiency-dependent method we tested led to clear differences between **Ψ **and **E**, which suggested that the spacing between amplification curves at a quantification or threshold cycle may not be a generally correct way of determining efficiency. As shown geometrically in figure 5, if two (modeled) amplification curves have the same slope at any given fluorescence, a sample initiated with a smaller number of template copies may yield a greater net slope (log_10_**E**) than the more concentrated sample (lines AC and DE are convergent). The horizontal spacing between amplification curves, from which **Ψ **is calculated, is established in an early cycle (length EC equals length DB) and does not reflect behavior over the entire amplification. The numerical results from the model in figure 5 are that **E **= 1.70 for amplification along segment DE and 1.74 along segment AC, but **Ψ **= 1.87 for the spacing between curves at the threshold (segment length EC). Actual amplification graphs might have more complex curvatures leading to convergence or divergence of spacing [24]. The model in figure 5 is meant only to explain and illuminate the potential for error between **Ψ **and **E**.

## Conclusions

The new method of qPCR analysis we have developed does not use any explicit or implicit measure of efficiency, and may therefore be immune to the problems outlined in the comparative methods of figures 2b, c, and 2d. We have applied this method successfully in control experiments with known amounts of DNA template, and also in cDNA experiments that confirm patterns of up-regulation of gene expression after DEX treatment of acute lymphoblastic leukemia cell lines. The method yields an estimate of absolute initial copy number of template, and while it is more laborious and expensive than other methods because it requires the development of an internal control and the assembly of multiple samples, it has the virtue of generating accurate results. We suggest that the algorithm should be included as a standard feature of software packages, in qPCR instruments of the future.

## Authors' contributions

YH: Performed the experimental work described. RDM: Principal investigator in the study of glucocorticoid-evoked apoptosis in lymphoblastic leukemia cell lines. SM: Developed the computational methods described, and drafted the manuscript. All authors read and approved the final manuscript.

## Supplementary Material

Additional file 1 **Source code for the perl program Deconvolution**. This is a perl program that performs the deconvolution analysis described in the paper. Depending on the computer platform this text file may need to be named with a file extension of .pl or .cgi. Click here for file

Additional file 2 **Sample data file for use with the perl program Deconvolution**. This is a tab-delimited text file containing sample data, suitable for use with the program Deconvolution. Click here for file

Additional file 3 **Manual for the perl program Deconvolution**. This is a pdf file explaining how to use the program Deconvolution. Click here for file
