# Supplementary material for: Quantitative polymerase chain reaction analysis by deconvolution of internal standard
Source: BMC Mol Biol. 2010 Apr 29;11:30. doi: 10.1186/1471-2199-11-30 (PMC2877679; doi:10.1186/1471-2199-11-30)
Supplement: Additional file 3 — Manual for the perl program Deconvolution. This is a pdf file explaining how to use the program Deconvolution. [file 1471-2199-11-30-S3.DOC]

# Manual for the perl program deconvolution.pl

Platform and modules: This program was developed on a Macintosh platform running Perl 5.8.8 in a Terminal window. It has also been used on a PC platform running ActivePerl 5.10.0 (build 1003). The program makes use of three perl statistics modules that should be downloaded from http://www.cpan.org and installed:

**Statistics::Basic::Mean;**

(http://search.cpan.org/~jettero/Statistics-Basic-1.6601/lib/Statistics/Basic/Mean.pod)

**Statistics::Basic::Variance;**

(http://search.cpan.org/~jettero/Statistics-Basic-1.6601/lib/Statistics/Basic/Variance.pod)

**Statistics::LineFit;**

(http://search.cpan.org/~randerson/Statistics-LineFit-0.07/lib/Statistics/LineFit.pm)

Data file format:

Upon being started, the program deconvolution.pl asks the user

***What tab-delimited PCR data file would you like to open? sample.txt***

The data are introduced as text files with tab-delimited fields, which can be generated using a program such as Microsoft Excel, where the file is saved with a format of “Text (Tab delimited)”. A file sample.txt is included in these materials as an example.


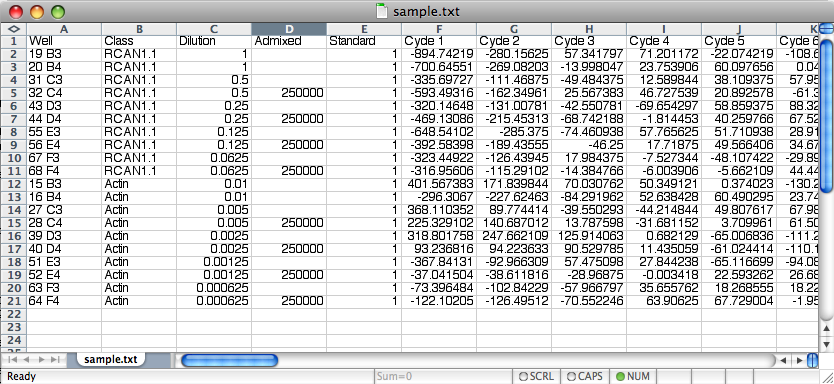


Records: The first row (record) of the file is ignored by the perl program and simply carries the titles of columns. Subsequent rows carry information and data on individual samples.

Fields (separated by tabs):

1. The sample name or well number for use in identification.
2. The class name of the sample. This allows groups (or classes) of samples to be analyzed together, for example if the file has data on several different amplicons.
3. The dilution of the unknown or experimental template. This is a number between 0 and 1, and for example, a dilution value of 0.5 indicates a 1:2 dilution of the original “1x” template
4. The amount of admixed amplicon DNA (in units of copies) as a control. The field is left blank if the sample is “neet”, meaning that it has no added control DNA.
5. An indication as to whether the sample should be included in the estimation of “1x” copy number as a “standard” (value of 1) or not (value of 0). If a 0 is placed in the field, the copy number in the sample will still be deduced for the final report using data from the “standard” samples, but will not be used in the optimization of correlation between Cq and log(A_0_).
6. The fluorescence data from cycle 1
7. The fluorescence data from cycle 2

… etc.

There is a pre-set limit of 100 cycles. However, this and other pre-sets can be changed by an expert user in the source code of the subroutine getPreferences.

After accepting the data file, the user is offered a choice of running the program in a manual mode:

***Do you want to take manual control of the optimization? (y/n)***

If the user does not enter “y” or “yes”, the program runs automatically and prints a final report on each class of samples. That “automatic” output will be discussed after the section titled “Manual Control” below.

Information is provided about the classification of samples, for example:

***The data in the file are classifed into 2 groups as follows:***

***Actin, with 6 neet and 4 admixed samples included in the standards***

***RCAN1.1, with 6 neet and 4 admixed samples included in the standards***

This information includes the name of the class (e.g. Actin, or RCAN1.1) taken from Field 2 in the data file, and the breakdown of types of samples (rows of data) that are included in the standards (i.e. they’re included if a 1 is entered in Field 5). A “neet” sample is one that consists solely of the unknown or experimental DNA template and has no admixed DNA control. An “admixed” sample is one that has both the experimental and control DNAs.

## Manual Control

Selection of thresholds: If the user requests manual control, then information is given about the maximum fluorescence values detected in the data, and the pre-set range of thresholds that can be tested (1% to 10% of the maximum). The user is asked if he or she wishes to change that range of values, for example:

***The fluorescence readings in your data have a maximum value of 2854563 units.***

***Based on your preferences, 50 threshold tests will be evenly spaced between 28545 and 285456 fluorescence units.***

***Would you like to change these settings? (y/n) y***

If the user replies “y” or “yes” then the lowest and highest threshold values may be set, and the number of tests that should be applied in that range (these are evenly spaced, once the range and number are established). For example, here the user requests 100 threshold tests between 10000 and 150000 fluorescence units:

Enter the following parameters...

***Lowest fluorescence value to test as a threshold: 10000***

***Highest fluorescence value to test as a threshold: 150000***

***Number of thresholds to test: 100***

***Let's double check your preferences...***

***The fluorescence readings in your data have a maximum value of 2854563 units.***

Based on your preferences, 100 threshold tests will be evenly spaced between 10000 and 150000 fluorescence units.

***Would you like to change these settings? (y/n)***

The program will repeat the process until the user is satisfied with the values entered.

Selection of estimation range (under manual control): If the user has requested manual control, then a rough estimate of copy number is provided (to seed the iteration process near the true value). The program is pre-set to suggest testing 100 geometrically spaced estimates starting at a minimum value determined by the structure of the data, and going up to 10 times over the rough estimate. For example:

***The estimate for copy number in the class Actin is 9.51e+07 copies in the undiluted sample. It is recommended that you consider 100 geometrically spaced estimates in the range 2.67e+07 to 9.51e+08 copies, a 35-fold range of values.***

***Would you like to change these settings? (y/n) y***

Upon replying “y”, or “yes”, the program queries the user for the range of estimates to test. Note that there is a lower limit to the estimate (2.67 x 10^7^ copies in this example), because there is a requirement that at least one admixed sample have an overall copy number less than the most concentrated neet sample.

***Enter the following parameters...***

***Lowest estimate to test: 1E7***

****** The number needs to be greater than 26666667 ******

***Lowest estimate to test: 3E7***

***Highest estimate value to test: 8E8***

***Number of estimates to test: 100***

***Let's double check your preferences...The estimate for copy number in the class Actin is 9.51e+07 copies in the undiluted sample. It is recommended that you consider 100 geometrically spaced estimates in the range 3.00e+07 to 8.00e+08 copies, a 26-fold range of values.***

***Would you like to change these settings? (y/n) n***

If there are additional classes of data, the program will query the user for the estimate ranges to apply, then will complete the first iteration and print a tab-delimited table of sample data and calculations. The best estimate (after the first iteration) is printed (in this case 1.011 x 10^8^ copies of actin, and this represents the copies of template in an undiluted “1x” sample. In this example of a sample file, it was annotated that the actin template was diluted 1:100 at its most concentrated (samples 15 B3 and 16 B4), meaning that those wells had approximately 1 x 10^6^ copies.

***Calculating ...***

***Results of optimization of Actin thus far (iteration #1)***

***The best estimate of Actin was 1.011e+08 copies***

Samples used in calculation:

***# Sample Dilution Admixed Est. A0 log(A0) Cq***

***11 15 B3 0.0100000 1.0110e+06 6.0047 13.7903***

***12 16 B4 0.0100000 1.0110e+06 6.0047 13.7503***

***13 27 C3 0.0050000 5.0548e+05 5.7037 14.7515***

***14 28 C4 0.0050000 2.5000e+05 7.5548e+05 5.8782 14.2795***

***15 39 D3 0.0025000 2.5274e+05 5.4027 15.9263***

***16 40 D4 0.0025000 2.5000e+05 5.0274e+05 5.7013 14.7646***

***17 51 E3 0.0012500 1.2637e+05 5.1016 16.8953***

***18 52 E4 0.0012500 2.5000e+05 3.7637e+05 5.5756 15.2797***

***19 63 F3 0.0006250 6.3184e+04 4.8006 18.0777***

***20 64 F4 0.0006250 2.5000e+05 3.1318e+05 5.4958 15.5950***

The linear correlation between Cq and log(A0) was strongest at a fluorescence threshold of 1.000e+04 units, (the tested range was 1.000e+04 to 1.486e+05 units)

***At this estimate and threshold, the parameters of the linear relationship between Cq and log(A0) were:***

Slope: -3.54726 0.001629 (Variance)

***Y-intercept: 35.05830 0.048990 (Variance)***

Linear correlation measures:

***R-squared: 0.998502***

***Mean Square Error: 0.00255***

***Do you wish to continue the optimization of the Actin class? (y/n)***

The table includes columns at left that indicate the row index number in the sample file, the sample name, the dilution, and the admixture of internal control. These are all taken from the sample file and re-printed for reference. Column 5: “Est. A0” is the estimated sum of the “unknown” and “known” components. For example, sample 28 C4 (in row 14) had a dilution of 0.005 and an admixture of 250000 copies, so the estimated initial copy number is

(0.005)(1.011 x 10^8^) + 2.5 x 10^5^ = 7.55 x 10^5^ copies.

Column 6 “log(A0)” is the base 10 logarithm of column 5. Column 7 “Cq” is the cycle number at which the fluorescence crosses a specific threshold, with interpolation based on a semilogarithmic graph. The specific threshold used is indicated below the table (in this example, 1 x 10^4^ fluorescence units). The threshold used in determining Cq is the one in the tested range that gave the most favorable linear correlation between the data in columns 6 and 7, with the optimization of the estimate performed simultaneously. That is, an estimate of 1.011 x 10^8^ copies (in the “1x” undiluted template) and a fluorescence threshold of 1 x 10^4^ units, taken together, generate the best correlation between log(A0) and Cq. The first iteration may be over a broad range of estimates, just to get a rough measure, and the user may conduct additional iterations over a more narrow range to search for a higher correlation.

After the user indicates that he or she does not wish to continue optimization, the next class of sample is addressed (if there is one). In the example file of sample.txt, the RCAN1.1 data are presented next (the classes are analyzed alphabetically).

## Automatic control

If manual control is not selected, the program does repeated iterations until a pre-set precision (±1%) or limit on iterations (10) is reached. These presets can be changed in the source code if desired (see subroutine: getPreferences). For example, here is the output from automatic optimization of the sample.txt file. The actin data were put through three iterations (with narrowing of the range) and the RCAN1.1 data were put through two iterations.

***Optimization of Actin is complete, having reached a precision of +/- 0.027%***

***Final results for Actin (after 3 iterations)***

***Iteration 1: tested range was 2.667e+07 to 9.103e+08 copies***

***Iteration 2: tested range was 9.977e+07 to 1.071e+08 copies***

***Iteration 3: tested range was 1.045e+08 to 1.046e+08 copies***

***The best estimate of Actin was 1.045e+08 copies***

***Samples used in calculation:***

***# Sample Dilution Admixed Est. A0 log(A0) Cq***

***11 15 B3 0.0100000 1.0455e+06 6.0193 15.3371***

***12 16 B4 0.0100000 1.0455e+06 6.0193 15.2363***

***13 27 C3 0.0050000 5.2273e+05 5.7183 16.3176***

***14 28 C4 0.0050000 2.5000e+05 7.7273e+05 5.8880 15.8348***

***15 39 D3 0.0025000 2.6137e+05 5.4173 17.5430***

***16 40 D4 0.0025000 2.5000e+05 5.1137e+05 5.7087 16.3272***

***17 51 E3 0.0012500 1.3068e+05 5.1162 18.4924***

***18 52 E4 0.0012500 2.5000e+05 3.8068e+05 5.5806 16.8719***

***19 63 F3 0.0006250 6.5342e+04 4.8152 19.6899***

***20 64 F4 0.0006250 2.5000e+05 3.1534e+05 5.4988 17.1972***

***The linear correlation between Cq and log(A0) was strongest at a fluorescence threshold of 2.855e+04 units, (the tested range was 2.855e+04 to 2.803e+05 units)***

***At this estimate and threshold, the parameters of the linear relationship between Cq and log(A0) were:***

***Slope: -3.63147 0.003210 (Variance)***

***Y-intercept: 37.14171 0.099028 (Variance)***

***Linear correlation measures:***

***R-squared: 0.998134***

***Mean Square Error: 0.00333***

***Optimization of RCAN1.1 is complete, having reached a precision of +/- 0.515%***

***Final results for RCAN1.1 (after 2 iterations)***

***Iteration 1: tested range was 2.667e+05 to 1.028e+07 copies***

***Iteration 2: tested range was 1.166e+06 to 1.255e+06 copies***

***The best estimate of RCAN1.1 was 1.216e+06 copies***

***Samples used in calculation:***

***# Sample Dilution Admixed Est. A0 log(A0) Cq***

***1 19 B3 1.0000000 1.2163e+06 6.0851 15.6485***

***2 20 B4 1.0000000 1.2163e+06 6.0851 15.6856***

***3 31 C3 0.5000000 6.0817e+05 5.7840 16.9370***

***4 32 C4 0.5000000 2.5000e+05 8.5817e+05 5.9336 16.2530***

***5 43 D3 0.2500000 3.0408e+05 5.4830 17.8627***

***6 44 D4 0.2500000 2.5000e+05 5.5408e+05 5.7436 17.0044***

***7 55 E3 0.1250000 1.5204e+05 5.1820 18.9243***

***8 56 E4 0.1250000 2.5000e+05 4.0204e+05 5.6043 17.4614***

***9 67 F3 0.0625000 7.6021e+04 4.8809 19.9640***

***10 68 F4 0.0625000 2.5000e+05 3.2602e+05 5.5132 17.7009***

***The linear correlation between Cq and log(A0) was strongest at a fluorescence threshold of 5.937e+04 units, (the tested range was 2.855e+04 to 2.803e+05 units)***

***At this estimate and threshold, the parameters of the linear relationship between Cq and log(A0) were:***

***Slope: -3.54317 0.000019 (Variance)***

***Y-intercept: 37.29037 0.000534 (Variance)***

***Linear correlation measures:***

***R-squared: 0.997772***

***Mean Square Error: 0.00376***

The automatic operation generates results quickly, but may not yield exactly the same correlation or estimate as manual operation. Using different ranges of estimates and thresholds may give results that differ by a few percent, as in the examples above, and this is expected.

For additional information, contact: Stan Metzenberg, Professor of Biology, California State University, 18111 Nordhoff St., Northridge CA 91330-8303. email: stan.metzenberg@csun.edu

Any corrected or improved versions of the program will be made available at http://www.escience.ws/deconvolution
